# Risk factors for lymphatic filariasis and mass drug administration non-participation in Mandalay Region, Myanmar

**DOI:** 10.1186/s13071-021-04583-y

**Published:** 2021-01-22

**Authors:** Benjamin F. R. Dickson, Patricia M. Graves, Ni Ni Aye, Thet Wai Nwe, Tint Wai, San San Win, Myint Shwe, Janet Douglass, Peter Wood, Kinley Wangdi, William J. McBride

**Affiliations:** 1 College of Medicine & Dentistry, Division of Tropical Health and Medicine, James Cook University, Cairns, QLD Australia; 2 College of Public Health, Medical and Veterinary Sciences, Division of Tropical Health and Medicine, James Cook University, Cairns, QLD Australia; 3 James Cook University and World Health Organization Collaborating Centre for Vector-Borne and Neglected Tropical Diseases, Townsville, QLD Australia; 4 Vector Borne Disease Control Unit, Ministry of Health and Sport, Naypyitaw, Myanmar; 5 Regional Vector Borne Disease Control Unit, Ministry of Health and Sport, Mandalay, Myanmar; 6 World Health Organization, Yangon, Myanmar; 7 General Practitioner, Mandalay, Myanmar; 8 Department of Global Health, Research School of Population Health, ANU College of Health & Medicine, The Australian National University, Canberra, ACT Australia

**Keywords:** Lymphatic filariasis, Risk factors, Myanmar, Asia, Infection, Hydrocoele, Mass drug administration, Coverage, Compliance, Participation

## Abstract

**Background:**

Myanmar commenced a lymphatic filariasis (LF) elimination programme in 2000. Whilst the country has made considerable progress since then, a number of districts have demonstrated persistent transmission after many rounds of mass drug administration (MDA). The causes of unsuccessful MDA have been examined elsewhere; however, there remains little information on the factors that contribute in Myanmar.

**Methods:**

We conducted an analysis of factors associated with persistent infection, LF-related hydrocoele and MDA participation in an area with ongoing transmission in 2015. A cross-sectional household survey was undertaken in 24 villages across four townships of Mandalay Region. Participants were screened for circulating filarial antigen (CFA) using immunochromatographic tests and, if positive, for microfilaria by night-time thick blood slide. Individuals 15 year and older were assessed for filariasis morbidity (lymphoedema and, if male, hydrocoele) by ultrasound-assisted clinical examination. A pre-coded questionnaire was used to assess risk factors for LF and for non-participation (never taking MDA). Significant variables identified in univariate analyses were included in separate step-wise multivariate logistic regressions for each outcome.

**Results:**

After adjustment for covariates and survey design, being CFA positive was significantly associated with age [odds ratio (OR) 1.03, 95% CI 1.01–1.06), per year], male gender (OR 3.14, 1.27–7.76), elevation (OR 0.96, 0.94–0.99, per metre) and the density of people per household room (OR 1.59, 1.31–1.92). LF-related hydrocoele was associated with age (OR 1.06, 1.03–1.09, per year) and residing in Amarapura Township (OR 8.93, 1.37–58.32). Never taking MDA was associated with male gender [OR 6.89 (2.13–22.28)] and age, particularly in females, with a significant interaction term. Overall, compared to those aged 30–44 years, the proportion never taking MDA was higher in all age groups (OR highest in those < 5 years and > 60 years, ranging from 3.37 to 12.82). Never taking MDA was also associated with residing in Amarapura township (OR 2.48, 1.15–5.31), moving to one’s current village from another (OR 2.62, 1.12–6.11) and ever having declined medication (OR 11.82, 4.25–32.91). Decreased likelihood of never taking MDA was associated with a higher proportion of household members being present during the last MDA round (OR 0.16, 0.03–0.74) and the number visits by the MDA programme (OR 0.69, 0.48–1.00).

**Conclusions:**

These results contribute to the understanding of LF and MDA participation-related risk factors and will assist Myanmar to improve its elimination and morbidity management programmes. 
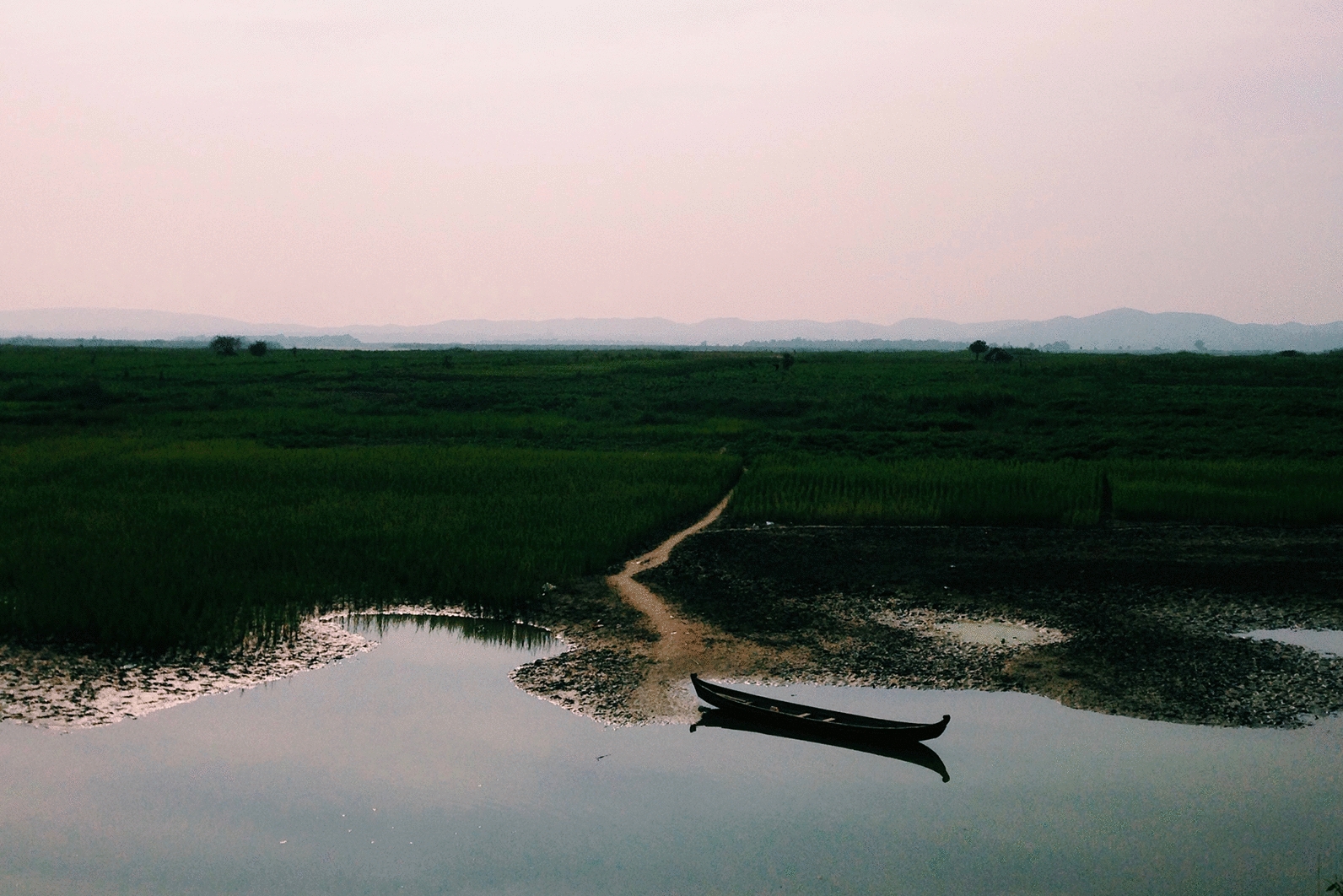

## Background

Lymphatic filariasis (LF) remains a major cause of permanent disability in tropical and sub-tropical countries [1]. Chronic infection with filarial worms causes lymphatic dysfunction leading to the progressive and irreversible swelling of the limbs and genitals. This results in substantial disability, discomfort, social stigma and economic disadvantage.

In response, the World Health Organisation (WHO) established the Global Programme to Eliminate LF in 2000, which is based on two intervention pillars. The first is to interrupt the transmission of LF through mass drug administration (MDA) of an annual dose of anti-filarial medications to at-risk populations [1]. The medications include albendazole plus either diethylcarbamazine (DEC) or ivermectin and are administered for a minimum of 5 consecutive years. The second component is to alleviate the suffering of those with existing LF-related disease through targeted management programmes.

Worldwide, the WHO South-East Asian Region has the highest burden of LF [1, 2]. Myanmar (formerly Burma) is one of the most affected countries in this region, with 41 million people (80% of the population) at risk [3, 4]. Filariasis in the country is distributed predominantly in the central and western dry zones, where baseline (pre-MDA) prevalence was 20 to 30% [3–6]. The parasite *Wuchereria bancrofti* is the sole reported cause of LF in the country where it is transmitted by the mosquito *Culex quinquefasciatus* [4, 7].

Myanmar commenced an MDA programme with DEC and albendazole in 2001. Since then, the country has made considerable progress toward the elimination of LF, but has also faced a number of challenges [3–5]. Medication supply and financial issues, as well as concerns over the reported incidence of serious adverse reactions, led to the interruption of a number of MDA rounds at both the national and regional levels [3, 8]. Whilst treatment coverage across the country has reportedly been high (60.0% to 100%), actual participation may have been much lower in some areas [3, 5, 9, 10].

Mass drug administration rounds have led to considerable reductions in LF prevalence in many areas of Myanmar [3–5]. A number of districts, however, have demonstrated persistent transmission despite many rounds of MDA, suggesting that the programme there had not been fully successful [3–5, 9, 10]. A number of reasons have been shown to account for the failure of MDA rounds to interrupt transmission in other countries [11, 12]. These include programmatic factors (the delivery and distribution of medication to eligible participants) or those relating to individual compliance (eligible participants taking the offered medication). Whilst there are common trends, the combination of factors that undermines success varies from region to region. Determining the causes of persistent transmission specific to Myanmar is crucial to ensuring effective elimination efforts.

Recently, an independent survey was conducted in four townships of Mandalay Region that had demonstrated ongoing transmission [9]. It found that 20% of the population had not participated in any of the six rounds of MDA. Of those who had, medication had only been taken on average 2.73 times. While this study provided an accurate estimate of LF burden and MDA participation, there remains no data on the potential causes of ongoing transmission or low medication uptake in Myanmar. We therefore conducted an analysis to determine the factors associated with persistent infection and non-participation (never taking MDA) in the country. Never taking MDA could be due to a number of factors, including lack of access (the programme did not reach the person to offer it), absence during MDA rounds, ineligibility due to age < 2 years, severe chronic illness or pregnancy, or active refusal.

## Methods

### Study setting

Myanmar is a lower-middle-income country in Southeast Asia which had a population of 51 million at the 2014 census [13]. The country is administratively divided into a union territory (Naypyitaw), seven states and seven regions. These 15 administrative areas are further divided into districts, townships, cities, towns, wards, village tracts and villages.

Mandalay Region is situated in the low-lying and dry central plains of Myanmar. The majority of its population (6 million in 2014) live rurally in villages, where the predominant occupation is farming [13]. The region’s capital, Mandalay, is the second largest city in Myanmar [13].

The study area included four of townships in the Mandalay Region (Amarapura, Patheingyi, Tada-U and Wundwin) where the prevalence of LF had been historically high [3, 14]. Patheingyi and Amarapura lie on the outskirts of Mandalay City, whilst Tada-U and Wundwin are located more rurally to the south. Amarapura, Patheingyi and parts of Tada-U Township lie close to the Irrawaddy River and its tributaries, whilst the Samon River passes through Wundwin. Amarapura also lies adjacent to the large Taung Tha Man Lake. A map of the study area is presented in Dickson et al. [9].

### Study design

A cross-sectional household survey was conducted between February and March 2015 to assess the prevalence of LF infection and disease, participation in the MDA programme and risk factors for LF and never taking MDA. The study methodology has been reported previously alongside the results for LF prevalence and MDA participation [9].

In brief, a two-stage random cluster sampling method was used to select households from 24 villages across the four townships. Consenting household members were screened for LF infection, examined for LF-related morbidity and completed a risk-factor questionnaire.

- Infection screening: Individuals 1 year and older were tested for circulating filarial antigen (CFA) (signifying current infection) using Binax Now Filariasis Immunochromatographic test (ICT) cards (Alere Inc, MA, USA) on finger-prick blood samples. Individuals who were antigen positive had a further 60 μl of finger-prick blood taken between 2200 and 0200 and applied to duplicate microscope slides using the three-line technique to assess for microfilaraemia (Mf).
- Clinical examination: Under the supervision of medical doctors, trained health workers examined participants aged 15 years and older for the presence of acute and chronic filariasis-related morbidity. Suspected cases of hydrocoele were confirmed using ultrasound (Sonosite M-Turbo, Bothwell, WA, USA).
- Risk factor questionnaire and GPS mapping: A pre-coded questionnaire was used to assess participation in the MDA programme as well as factors associated with LF infection, disease and never taking MDA. Prior to data collection, the questionnaire was translated into the Myanmar language (Burmese) and pre-tested with staff of the Vector Borne Disease Control Unit. Trained health workers completed a brief questionnaire with each participant (Additional file 1) followed by a more detailed questionnaire with the household head (Additional file 2). Direct observation was used to verify respondent answers where possible. A global positioning device was used to record the location and elevation of each household.

### Data analysis

Independent variables: Data were collected on paper forms in the Myanmar language (Burmese) and entered into Excel format before transfer to STATA. The distance between households and large bodies of water was calculated in ArcMap 10.5 (ESRI, Redlands, CA). An electronic map of inland water bodies in shapefile format was obtained from Global Administrative Areas database (http://www.gadm.org/country). The distance between households and water bodies was then calculated using the Near function of the Proximity Tool. The shortest distance was determined using the planar (flat earth) method. An LF knowledge score was created from survey questions to assess the relationship between knowledge of LF and MDA participation. Participants received a point each if they had heard of LF and then if they correctly identified LF’s mode of transmission, symptoms and treatment, giving a maximum possible score of four points.

Univariate analyses: Statistical analysis was completed using STATA version 14.2 (StataCorp, Colleg Station, TX, USA). First, a series of univariate analyses were completed to assess the association between potential risk factors for each of the three outcomes: infection (CFA positive), LF related-hydrocoele and never taking MDA medication. A risk factor analysis for lymphoedema could not be undertaken as no cases were identified in the sample. Microfilaria positivity was not included as an outcome variable because there were a small number of Mf positive individuals and the results were collinear with CFA positivity.

The ‘svy’ prefix command was used to adjust for clustering effect and weight for sampling probability. *T*-tests, *χ*^2^ and logistic regression were used to compare variables and generate odds ratios adjusted for survey design. Independent variables with a *p*-value < 0.2 were included in the multivariate analysis. Circulating filarial antigen positivity and hydrocoele demonstrated a linear association with age, so age by year was used. In contrast, the association between never taking MDA and age had a bimodal distribution, so age by quintile was used. Total household income data demonstrated a skewed distribution, so the log of income was used to normalise its distribution.

Multivariate analyses: Next, separate backward multivariate logistic regression analyses were completed for each of the three outcomes with adjustment for survey design. The variable ‘Township’ was added at the end of the analyses to assess whether there were additional unmeasured fixed effects associated with township that had not been accounted for. Risk factors with a *p*-value < 0.05 were considered significant. Participants with incomplete data were excluded from analyses.

## Results

### Characteristics of the study population

A total of 450 households in 24 villages were sampled. Of those, 20 households (4.4%) were excluded: 15 were absent and 5 declined to participate. From the remaining 430 households, 1014 individuals participated in the study. The median age of participants was 36 years [interquartile range (IQR): 30, range: 1–86]. Significantly more females than males were included in the sample (63.7% *vs* 36.3%, *p* < 0.001) with no significant difference in age between genders. The median number of inhabitants per household was four (IQR: 3, range: 1–12) with an average household monthly income of 120,000 Myanmar kyat (85 USD) (IQR: 120,000, range: 5,000 to 1,500,000).

### LF infection prevalence

Forty-six of the 1001 participants tested for infection by CFA were positive (crude prevalence: 4.6%, adjusted for age, gender and survey design: 2.63%). The median age of infected individuals was 46 years (IQR: 23, range 8–81 years). A cluster of five villages in Amarapura and Tada-U townships had a notably higher prevalence of infection compared to the remaining villages (mean 13.32% *vs* 1.14%). Microfilariae were found in 39.02% of antigen positive individuals, representing an overall adjusted Mf prevalence estimate of 1.03% (assuming all antigen negative persons were also Mf negative).

### Hydrocoele prevalence

Fifteen of the 269 men aged over 15 years who were examined had an LF-related hydrocoele (crude prevalence 5.58%, adjusted for age and survey design 2.78%). The median age of males suffering from an LF-related hydrocoele was 55 years (IQR: 12, range: 48–71 years). The highest prevalence occurred in three villages in Amarapura Township where adjusted prevalence ranged from 21.53 to 36.81%.

### MDA programme participation

Ninety-five per cent of households reported ever being visited by the MDA programme. Of these, the mean number of visits was 2.59. Ninety per cent of household members reported being present during the last MDA round in 2014. Eighty per cent of participants reported taking MDA medication at least once—in other words, 20% were systematic non-takers who had never taken MDA. Of those who had ever taken MDA, the mean number of times medication had been consumed was 2.73. Ten per cent of participants had actively declined to take MDA medication (despite it being offered) on at least one occasion. The most common reasons reported were a fear of side effects and a perception that co-morbidities were a contraindication.

### Risk factors for LF infection

Table 1 outlines the univariate analysis of factors associated with LF infection (by CFA) adjusted for survey design (i.e. weighted for sampling probability and adjusted for clustering). Infection was positively associated with age (by year), male gender, household monthly income, residing in Amarapura township, the absence of screens/glass on household windows, the number of people per household room and having moved to one’s current village. Infection was also positively associated with never taking MDA medication as well as not taking it in the last year. Infection was negatively associated with elevation (per metre) and distance to the nearest body of water (per km).

**Table 1:** Univariate logistic regression analysis of association between infection (measured by CFA^a^) and individual/household risk factors; weighted and adjusted for survey design (*n* = 1001)

Risk factor	Total (*n*)	CFA positive (%)^b^	CFA positive		
			aOR^b^	95% CI^c^	*P*
Individual risk factors					
Age (per year)	997	–	1.03	1.01–1.05	*0.011*
Gender	1001				
Male	365	4.76	2.34	1.03–5.36	*0.044*
Female	636	2.09			
Occupation	975				
None/home duties	257	2.84	1.01	0.27–3.82	0.985
Student	160	0.78	0.27	0.02–3.20	0.286
Manual labour	519	4.00	1.45	0.35–5.97	0.596
Other (ref)	39	2.80			
Work environment	947				
Outdoor	325	3.26	1.08	0.47–2.47	0.853
Indoor or mixed	622	3.03			
Moved to current village	992				
Yes	148	8.13	3.46	1.45–8.25	*0.007*
No	844	2.49			
Slept under a bednet last night	996				
Yes	907	2.75	0.46	0.10–2.07	0.298
No	89	5.76			
Never taken MDA medication	994				
Yes	188	7.55	3.67	1.51–8.93	*0.006*
No	806	2.18			
No. times MDA taken	985	–	0.78	0.57–1.08	0.133
0 (ref)	188	7.55			
1–2	336	2.92	0.37	0.12–1.16	0.085
3–4	273	0.57	0.07	0.01–0.37	*0.003*
5–6	188	4.40	0.56	0.20–1.62	0.273
Did not take MDA last year	994				
Yes	237	7.18	3.80	1.57–9.17	*0.005*
No	757	2.00			
Household risk factors					
Township	1001				
Amarapura	297	9.07	9.96	2.98–33.29	*0.001*
Patheingyi	113	2.61	2.68	0.24–29.54	0.405
Tada-U	267	3.38	3.49	0.87–13.94	0.075
Wundwin (ref)	324	0.99			
Log household monthly income	965	–	1.89	1.30–2.75	*0.002*
Highest level of education	993				
None/primary	325	3.38	7.44	0.82–67.34	0.072
Secondary	528	3.81	8.43	0.95–74.90	0.055
Tertiary (ref)	140	0.47	1.00		
Elevation (by metre)	997	–	0.95	0.92–0.99	*0.011*
Distance to nearest water body (per km)	989	–	0.91	0.85–0.98	*0.011*
No screens or glass on windows	994				
Yes	373	6.43	3.52	1.32–9.44	*0.015*
No	621	1.92			
No. people in household	997	–	1.24	0.96–1.60	0.092
No. people per bednet (ratio)	987	–	1.23	0.99–1.53	0.065
No. of people per room (ratio)	965	–	1.60	1.35–1.89	< *0.001*

^a^Circulating filarial antigen

^b^Adjusted for survey design

^c^95% confidence interval

Italics values indicate *P* < 0.05

Table 2 demonstrates the factors significantly associated with LF infection following adjusted multivariate logistic regression. Forty-nine of the 1001 participants tested for infection were excluded from the final multivariate model due to missing data. Infection was positively associated with age (per year), male gender and the number of people per household room. Infection was negatively associated with elevation (per metre).

**Table 2:** Multivariate logistic regression analysis of association between infection (measured by CFA^a^) and individual/household risk factors; weight and adjusted for survey design (*n* = 952)

Risk factor	aOR^b^	95% CI^c^	*P*
Individual risk factors			
Age (per year)	1.03	1.01–1.06	*0.003*
Male gender	3.14	1.27–7.76	*0.015*
Never taken MDA medication	2.15	0.73–6.32	0.154
Household risk factors			
Elevation (m)	0.96	0.94–0.99	*0.019*
No. of people per room (ratio)	1.59	1.31–1.92	< *0.001*

^a^Circulating filarial antigen

^b^Adjusted for survey design

^c^95% confidence interval

Italics values indicate *P* < 0.05

### Risk factors for LF-related hydrocoele

Table 3 shows the univariate association between LF-related hydrocoele and potential risk factors. Hydrocoele was positively associated with age (by year) and residing in Amarapura Township and negatively associated with working in an outdoor environment. A relationship between hydrocoele, frequency of bathing and shoe wearing was hypothesised; however, there was insufficient variance in responses to assess the association.

**Table 3:** Univariate logistic regression analysis of association between LF-related hydrocoele and individual/household risk factors; weighted and adjusted for survey design (*n* = 269)

Risk factor	Total		Proportion with hydrocoele (%)^a^	Hydrocoele present		
				aOR^a^	95% CI^b^	*P*
Individual risk factors						
Age (by year)		268	–	1.05	1.03–1.08	< *0.001*
Occupation		265				
None/home duties		32	10.45	1.85	0.17–2.82	0.603
Student		2	0	–		
Manual labour		213	3.32	0.54	0.04–6.89	0.625
Other (ref)		18	5.93	1.00		
Work environment		258				
Outdoor		145	0.85	0.20^c^	0.05–0.72	*0.014*
Indoor or mixed		113	7.29			
Moved to current village of residence		268				
Yes		42	7.68	2.14	0.45–10.24	0.327
No		226	3.75			
CFA^d^ positive		269				
Yes		19	12.25	3.65	0.81–16.38	0.088
No		250	3.69			
Microfilariae positive^e^		266				
Yes		7	18.18	5.39	0.83–35.04	0.076
No		259	3.96			
Never taken MDA medication		269				
Yes		56	4.66	1.15	0.22–5.98	0.859
No		213	4.07			
Household risk factors						
Township		269				
Amarapura		59	13.92	11.12	1.85–66.89	*0.011*
Patheingyi		27	8.02	5.98	0.40–89.48	0.184
Tada-U		64	1.73	1.21	0.08–17.21	0.884
Wundwin (ref)		119	1.44	1.00		
Log household monthly income				1.43	0.77–2.64	0.241
Highest HH education		268				
None/primary		81	2.74	0.26	0.03–2.78	0.253
Secondary		150	3.29	0.32	0.04–2.41	0.253
Tertiary (ref)		37	9.72	1.00		
Drinking water type (by WHO classification)		268	–	1.01	0.41–2.47	0.981
Sanitation type (by WHO classification)		269	–	0.26	0.04–1.63	0.143
Elevation (by metre)		269	–	0.98	0.96–1.01	0.148
Distance to nearest water body (per km)		267	–	0.95	0.89–1.01	0.075
No. of people per HH room (ratio)		259	–	1.35	0.92–1.98	0.116

^a^Adjusted for survey design

^b^95% confidence interval

^c^Unadjusted values given due to study stratum with single sampling unit

^d^Circulating filarial antigen

^e^All CFA negative individuals assumed to be microfilariae negative

Italics values indicate *P* < 0.05

Table 4 summarises the significant factors associated with LF-related hydrocoele following multivariate logistic regression. All but 1 of the 269 men examined for hydrocoele were included in the multivariate model. Hydrocoele was positively associated with age (by year) and residing in Amarapura Township.

**Table 4:** Multivariate logistic regression analysis of association between LF-related hydrocoele and individual/household risk factors; adjusted for survey design (*n* = 268)

Risk factor	aOR^a^	95% CI^b^	*P*
Individual risk factors			
Age (by year)	1.06	1.03–1.09	*0.001*
Household risk factors			
Township			
Amarapura	8.93	1.37–58.32	*0.024*
Patheingyi	8.17	0.50–134.56	0.134
Tada-U	1.56	0.11–21.75	0.755
Wundwin (ref)	1.00		

^a^Adjusted for survey design

^b^95% confidence interval

Italics values indicate *P* < 0.05

### Risk factors for never taking MDA medication

Table 5 shows the univariate analysis of potential factors associated with never taking MDA medication, adjusted for survey design. Never taking MDA was positively associated with male gender, moving to one’s current village, declining offered MDA medication, residing in Amarapura and Tada-U Townships and the number of people per household room. Never taking medication was negatively associated with knowledge of LF, being visited by the MDA programme and the number of times visited. Since never taking MDA occurred more often in those who declined medication and had a lower knowledge of LF, a side analysis of the relationship between these two independent variables was conducted. Declining offered medication was significantly less likely in those with greater LF knowledge (OR = 0.68, *p* = 0.025).

**Table 5:** Univariate logistic regression analysis of association between never taking MDA medication and individual/household risk factors; weighted and adjusted for survey design (*n* = 1007)

Risk factor	Total (n)	Proportion that had never taken MDA medication (%)	Never taken MDA medication		
			aOR^a^	95% CI^b^	*P*-value
Individual risk factors					
Age group (years)	1003				
0–14	177	18.36	1.49	0.74–3.03	0.254
15–29	201	20.69	1.73	0.93–3.21	0.079
30–44 (ref)	273	13.10	1.00		
45–59	221	13.64	1.05	0.53–2.07	0.888
60+	131	22.57	1.93	0.89–4.21	0.092
Gender	1007				
Male	366	21.31	1.61	1.10–2.35	*0.017*
Female	641	14.43			
Occupation	986				
None/home duties	263	15.61	0.55	0.20–1.53	0.241
Student	163	17.29	0.63	0.17–2.36	0.472
Manual labour	520	16.86	0.61	0.23–1.59	0.296
Other (ref)	40	25.04	1.00		
Moved to their current village of residence	1000				
Yes	146	27.96	2.08	1.06–4.07	*0.034*
No	854	15.74			
Ever declined MDA medication	935				
Yes	74	52.36	9.18	2.43–34.65	*0.002*
No	861	10.70			
Household risk factors					
Township	1007				
Amarapura	304	20.22	1.81	1.08–3.03	*0.027*
Patheingyi	111	20.29	1.82	0.37–8.80	0.443
Tada-U	268	20.52	1.84	1.10–3.08	*0.022*
Wundwin (ref)	324	12.30	1.00		
Log household monthly income	970	–	1.52	0.99–2.34	0.055
Highest level of education	999				
None/primary	326	16.83	1.91	0.76–4.82	0.163
Secondary	531	19.75	2.32	0.85–6.31	0.095
Tertiary (ref)	142	9.59	1.00		
Literacy of household head	994				
Illilterate	84	15.18	0.87	0.34–2.21	0.751
Partially literate	54	22.64	1.41	0.55–3.67	0.460
Literate	856	17.15	1.00		
Knowledge of LF (by score, *n* of 4)	959	–	0.66	0.46–0.94	*0.024*
Visited by MDA programme	997				
Yes	939	14.30	0.07	0.03–0.18	< *0.001*
No	58	69.30			
Total number of MDA programme visits (by *n* of 6)	961	–	0.49	0.33–0.73	*0.001*
Proportion of household present during last MDA (ratio)	900	–	0.28	0.06–1.34	0.107
No. of people per room (ratio)	970	–	1.22	1.04–1.43	*0.017*

^a^Adjusted for survey design

^b^95% confidence interval

Italics values indicate *P* < 0.05

Table 6 illustrates the factors significantly associated with never taking MDA following multivariate analysis, with adjustment for survey design. Of the 1007 participants who self-reported MDA participation, 163 were excluded from the final multivariate model as a result of missing data. Never taking MDA was positively associated with extremes of age (< 15 years and ≥ 60 years), male gender, moving to one’s current village of residence, ever declining MDA medication and residing in Amarapura Township. It was negatively associated with the number of visits by the MDA programme and the proportion of household members present during the last MDA.

**Table 6:** Multivariate logistic regression analysis of association between never taking MDA medication and individual/household risk factors; weighted and adjusted for survey design (*n* = 844)

Risk factor	Without age–gender interaction term			With age–gender interaction term		
	aOR^a^	95% CI	*P*	aOR^b^	95% CI	*P*
Individual risk factors						
Age group (years)						
0–14	2.66	1.08–6.62	*0.035*	4.80	1.40–16.42	*0.015*
15–29	1.83	0.91–3.70	0.089	3.78	1.23–11.66	*0.023*
30–44 (ref)	1.00			1.00		
45–59	1.28	0.64–2.55	0.476	3.37	1.0–10.32	*0.035*
60+	4.03	1.53–10.59	*0.007*	12.82	3.34–49.18	*0.001*
Male gender	1.88	1.10–3.23	*0.024*	6.89	2.13–22.28	*0.002*
Age-gender (male) interaction						
0–14				0.38	0.07–2.10	0.253
15–29				0.29	0.07–1.16	0.078
30–44 (ref)				1.00		
45–59				0.17	0.04–0.68	*0.015*
60+				0.10	0.02–0.45	*0.004*
Moved to current village of residence	2.57	1.08–6.15	*0.035*	2.62	1.12–6.11	*0.027*
Ever declined MDA medication	10.52	3.86–28.72	< *0.001*	11.82	4.25–32.91	< *0.001*
Household risk factors						
Township						
Amarapura	2.41	1.14–5.12	*0.024*	2.48	1.15–5.31	*0.022*
Patheingyi	2.07	0.69–6.17	0.183	1.89	0.63–5.68	0.246
Tada-U	0.55	0.24–1.23	0.136	0.55	0.24–1.26	0.149
Wundwin (ref)	1.00			1.00		
Total number of MDA programme visits (by *n* of 6)	0.67	0.46–0.97	*0.033*	0.69	0.48–1.00	*0.047*
Proportion of household present during last MDA (by ratio)	0.17	0.04–0.80	*0.026*	0.16	0.03–0.74	*0.021*

^a^Adjusted for survey design

^b^95% confidence interval

Italics values indicate *P* < 0.05

The proportion of those who had never taken MDA by age varied between genders (Additional file 3: Figure S1). To assess the influence of gender on age, an interaction term was added to the multivariate analysis (Table 6). The analysis indicated that there was significant interaction between age and gender, with the effect of age much more apparent in females. Females aged 30 to 44 years are most likely to have taken medication, whilst males of all ages and females < 30 years or > 44 years old were more likely to have never taken MDA.

## Discussion

This study has been the first to examine risk factors related to LF and MDA participation in Myanmar. It assessed three outcomes in an area with ongoing transmission after six rounds of MDA including: persistent infection (measured by CFA), never taking MDA medication and LF-related hydrocoele. It found that persistent infection was related to both individual demographics (age and gender) and household factors (altitude and density of persons per room). Never taking MDA medication was associated with both programme reach and individual compliance factors. Finally, LF-related hydrocoele was significantly associated with increasing age and residing in the historically endemic township of Amarapura.

Despite six rounds of MDA, infection remained significantly associated with baseline risk factors, which include host characteristics and exposure to infected vectors. The relationship between infection and increasing age found in this study is well established [15]. It is thought to relate to both increasing exposure to infected mosquitoes with age and the inefficient transmission of L3 larval stages from mosquitoes to humans [16].

The higher prevalence of LF infection amongst males found here is also well documented [15, 17]. Historically, it was suggested that this was the result of greater occupational exposure. However, the persistence of male predominance, following adjustment for occupation, outdoor work and MDA participation in this analysis, supports the hypothesis that there is also a biological basis for this difference [18–20].

Risk of infection was strongly associated with the number of people per household room, a marker of crowding. Entomological studies have shown that rooms with more inhabitants have an increased density of indoor *Culex* mosquitoes [21]. It is therefore hypothesised that crowding may lead to greater density of indoor *Culex* mosquitoes increasing transmission risk amongst household members. Efforts to reduce overcrowding, which are employed for other communicable diseases, may therefore also decrease LF transmission. Whilst a relationship between crowding and infection has not previously been documented, a link has been observed between household size and infection in children in Brazil, where *Culex* is also the vector [22]. When crowding was replaced by household size in our final model, there was a positive association, but it did not reach significance (*p* = 0.058, results not shown). This suggests that household size, as well as density, may predispose to infection in *Culex* regions, but this requires further investigation.

The absence of screens or glass on household windows was also associated with higher infection risk but lost significance in the multivariate analysis following adjustment for survey design. This may be due to sample heterogeneity, and that Amarapura Township had both a high infection prevalence and low number of screens/glass on windows. The relationship is nonetheless noteworthy, because studies in Africa have shown that windows and doors are the preferred entry point for *Culex* mosquitoes, and the addition of screens reduces their indoor density [23, 24]. Other variables related to night-time vector exposure, including bednet ownership and usage, also showed a protective association, but did not reach significance. This was probably because bednet ownership and usage were uniformly high across the study area. Although these associations did not reach significance in the final model, together they indicate that individuals may be infected whilst in their household but outside of their bednet. The addition of screens to household windows could therefore be an effective intervention to reduce LF transmission and deserves further study and trials.

An interesting finding was the association between small changes in elevation and infection risk. Whilst a negative association between elevation and infection risk is well documented, this typically occurs with larger variations in altitude [25, 26]. This is because at higher altitudes, atmospheric temperatures decrease, resulting in reduced mosquito survival and slower parasite development within the vector [27]. In this area, which is located within the central plains of Myanmar, altitude only ranged between 45 and 269 m above sea level. This would be insufficient to affect vector survival, but instead indicates low-lying households that may be closer to bodies of water. The distance from households to large bodies of water was also directly measured. Whilst significant in the univariate analysis, the variable lost significance when it replaced or was added alongside elevation in the final model. This could be because elevation more accurately captured subtle proximity to water such as smaller waterways, areas prone to flooding or households closest to water along a riverbank. These findings support earlier work in demonstrating the usefulness of elevation as an indirect marker for proximity to water and therefore infection risk in low-lying contexts [28].

A suggestive finding in this study was the association between persistent infection and never taking MDA. Whilst the relationship lost significance after adjustment for survey design in the multivariate analysis, possibly due to the clustering of those who had never taken MDA, it retained a strong positive effect (OR = 2.15). This relationship may be explained by both the mechanisms with which MDA medications control LF at the individual and community levels. The predominant effect of MDA is microfilaricidal, thereby reducing local transmission and the chance of new infection [29]. This is reflected in the study area, where villages with > 65% MDA participation had a mean CFA prevalence of 2.08% compared to 9.76% in villages with less participation. The secondary effect is macrofilaricidal, reducing the burden of adult worms and therefore eventually CFA [30]. At the individual level, this likely explains the lower weighted and adjusted prevalence in those who had participated in the MDA programme (1.60%) compared to those who had not (6.74%). The high prevalence of infection amongst ‘systematic non-takers’ supports studies elsewhere in highlighting their significant role as a reservoir for ongoing transmission in the community [31, 32].

Compared to the logical relationship between infection and never taking MDA medication, the association with the number of times MDA was taken was complex and non-intuitive. Relative to those who have never taken MDA, infection prevalence progressively reduced in those who took MDA a few or intermediate number of times, but then increased in those who took MDA five or six times (Table 1). All of the participants with infection who reported taking five to six rounds of MDA resided in Amarapura, the highest prevalence township (Additional file 4: Figure S2). These individuals may therefore have been more intensively targeted, at greater risk of re-infection or more familiar with MDA and thus more likely to give responses showing social desirability bias. Of note, the accuracy of self-reported MDA participation has not been validated beyond 1 year, so may also be prone to significant recall bias.

Understanding the causes of never taking MDA medication (i.e systematic non-participation) are crucial to improving control efforts, that is, distinguishing between programmatic deficiencies (not reaching households or visiting at inconvenient times) from individual reasons (such as refusal or perceived ineligibility). In the study area, never taking MDA was related to both programmatic reach (the proportion of targeted persons who are offered medication) and individual compliance (the proportion who take the offered medication).

The programmatic factors associated with never taking MDA suggested households were either not visited by the programme or the members of the household were absent at the time of the visit. While almost all (95%) surveyed households reported being visited by an MDA programme member at least once, the mean number of visits was only 2.59, with fewer visits predictive of never taking MDA. In the households that were visited, absenteeism further predisposed to never taking medication. Never taking MDA was less likely in persons from households with more members present during the last round.

The association between never taking MDA, age and gender may also relate to absenteeism. Males of all ages and females outside of the 30 to 44 year age group were more likely to have never taken MDA. In Mandalay Region, women are more likely than men to be household workers, with the greatest proportion of these women aged between 25 and 49 years [33]. This suggests that the programme may have more successfully reached these women because they were at home during the day compared to the others who were more likely to be absent.

The greater likelihood of never taking MDA in persons that had migrated from another village may also relate to programme reach. This finding was somewhat unexpected as we had no prior knowledge of the frequency, extent or origin of population movement. Domestic migration could be a cause of never taking medication for a variety of reasons, such as moving from non-endemic areas, missing rounds due to travel or non-registration at the current residence. A limitation of our study was that we did not ascertain the source of migration, which is likely to include both long-range migration from areas up-river as well as local movement between villages. Information on migration including its source and frequency should be further investigated in future studies together with information on length of residence in their village.

These findings corroborate those of Linn et al. who recently conducted a qualitative survey on barriers to MDA participation in Myanmar (unpublished) [34]. They interviewed members of the community and National Program to Eliminate LF (NPELF) in three townships with persistent transmission in Mandalay and the adjacent Magway and Sagaing Regions. They found community members were often unaware of previous rounds, suggesting they may have been missed. Of those who were aware of the previous rounds, a number stated they had not received medication because they were away. Meanwhile, drug distributors reported that financial, human resource and time limitations prevented them from fulfilling their duties and mopping-up recipients who were initially missed. The NPELF should therefore ensure that future rounds reach all households, are sufficiently resourced and are conducted at a time when household members are likely to be present. A strong emphasis should also be made to track down eligible recipients who were missed during the initial round.

In addition to programmatic reach, never taking MDA was also at least partly related to individual compliance in the study area. A notable proportion of the participants (8% of 935) reported that they had refused MDA at least once in the past. Of those, 52% had never taken MDA compared to only 11% among participants who had never actively refused medication. Thus, ever declining medication was strongly associated with never taking MDA. The most common reasons for declining MDA reported in both our study and that of Linn et al. were fear of side effects and a perception that co-morbidities were a contraindication [34]. Although adverse events are rare, community concern has hampered MDA in many countries and resulted in the postponement of the 2006 MDA round in the Mandalay Region [3].

Education initiatives regarding LF, the MDA programme and common misconceptions have been effective in improving MDA compliance [35–37]. These studies reported that greater LF knowledge reduced the likelihood of declining medication and non-participation, suggesting that these interventions would likely also be effective in Myanmar. The NPELF should consider efforts to improve community education and dispel myths to improve MDA participation.

Amarapura had significantly more individuals who had never taken MDA compared to the other townships in the study. Douglass et al. similarly found low participation in young people surveyed there in 2013 and 2014 [10]. This lower participation could not be accounted for by the programmatic reach and compliance factors assessed. Of the four townships, Amarapura is the most urban and had the highest average household income. Studies elsewhere have found that MDA participation can be more difficult to achieve in urban areas because of a lack of urban strategy, fewer peripheral health workers, poor health care infrastructure, the presence of unorganised settlements and large numbers of migrants [11]. Compared to other townships, Amarapura had the highest proportion of individuals who had moved from another village but a similar ratio of household members present during the last round and the highest number of NPELF visits. Within urban areas, studies have also found that higher income individuals can be harder to reach in surveys and are more likely to decline to participate in MDA programmes [11]. Amarapura had the lowest proportion of individuals who reported declining MDA medication. When income was assessed in a subgroup analysis of the township, however, there was a trend toward lower participation with higher income but it did not reach significance (cOR = 1.07; *p* = 0.642; aOR = 1.41; *p* = 0.080). The lower participation levels in Amarapura, therefore, may relate to its urban location, more mobile population and higher incomes, but this requires further analysis.

In addition to exploring potential causes for persistent infection and never taking MDA, this study also assessed risk factors for LF-related hydrocoele. The positive association found between age and hydrocoele has been well documented [15]. This is thought to be due to the progressive onset of lymphatic dysfunction with chronic filarial infection [38]. Hydrocoele was not found to be associated with CFA positivity following adjustment for survey design. Men with hydrocoele are often CFA negative, reflecting the fact that the active infection has often ceased by the time hydrocoeles develop. The higher risk of hydrocoele in Amarapura probably relates to the historically high infection prevalence there. These findings underscore the importance of reducing infection to prevent the development of LF-related hydrocoele.

The study results reported should be interpreted in light of some limitations. First, more females than males participated in the study. Whilst gender was adjusted for, it led to wider odds ratio confidence intervals. Second, data were missing from a number of risk factor variables (see total column in Tables 1, 3 and 5) leading to fewer numbers of participants in the final models for infection and never taking MDA. In addition to widening confidence intervals, it is possible this may have biased the results. Third, other potential environmental factors such as rainfall, temperature and population density were not included, which may have provided a more complete explanation of risk. Lastly, local health workers and members of the NPELF assisted with data collection. It is therefore possible that participants’ responses regarding MDA participation could have been biased towards ‘acceptable’ replies. The low levels of reported MDA participation, however, suggest this was unlikely.

The results of this study will assist Myanmar’s NPELF and contribute to the global understanding of LF risk factors. Knowledge of the factors associated with persistent infection and never taking MDA will help the National Programme to better allocate its resources in elimination efforts. Maximising MDA participation is critical as Myanmar transitions from the two-drug to three-drug MDA (ivermectin, diethylcarbamazine and albendazole, IDA) starting in 2020, since models predict that fewer rounds of IDA are needed to stop transmission [39]. To do this, our results suggest that emphasis should be placed on both improving reach, with particular attention to mopping-up missed participants, and compliance through educational initiatives. Since the findings reflect many common global reasons for poor MDA uptake and those of Linn et al., it is reasonable to infer that they could be generalisable to other regions of Myanmar. The identified infection risk factors may also apply to other countries where *Culex* is the vector. Finally, the identification of hydrocoele risk factors contributes to the understanding of LF morbidity and will help Myanmar to better target alleviation programmes.

## Conclusions

This population-representative study assessed risk factors for LF infection, disease and never taking MDA in an area of persistent LF transmission in Myanmar. It found that persistent LF infection was linked to both individual and household factors. In turn, never taking MDA was related to both programme reach and individual compliance factors. It also highlighted that hydrocoele risk was higher in those who were older and residing in the historically high prevalence township of Amarapura. These results will assist the NPELF to improve its MDA rounds and to better target its morbidity management programmes.

## Supplementary Information

**Additional file 1.** Individual participant survey in English and Myanmar languages.

**Additional file 2.** Household survey in English and Myanmar languages.

**Additional file 3: Figure S1.** Unadjusted proportion who never took MDA by age and gender.

**Additional file 4: Figure S2.** Unadjusted infection prevalence by number of times MDA medication taken and township.

## Data Availability

All data generated or analysed during this study are included in this published article and its Additional files.

## Abbreviations

LF: Lymphatic filariasis
MDA: Mass drug administration
WHO: World Health Organisation
NPELF: National Program to Eliminate LF
CFA: Circulating filarial antigen
Mf: Microfilaraemia
ICT: Immunochromatographic test
OR: Odds ratio
95%CI: 95% Confidence interval
IQR: Interquartile range
