# Supplementary material for: Risk factors for lymphatic filariasis and mass drug administration non-participation in Mandalay Region, Myanmar
Source: Parasit Vectors. 2021 Jan 22;14:72. doi: 10.1186/s13071-021-04583-y (PMC7821648; doi:10.1186/s13071-021-04583-y)
Supplement: Supplementary file 1 — Additional file 1. Individual participant survey in English and Myanmar languages. [file 13071_2021_4583_MOESM1_ESM.pdf]

<table>
<tr><td rowspan="2">နေ့စွဲ</td><td rowspan="2"></td><td rowspan="2">နာမည်
အတို</td><td rowspan="2"></td><td>မြို့နယ်</td><td colspan="2">ကျေးရွာ</td><td colspan="2">အိမ်ခြေ</td><td colspan="2">လူဦးရေ</td></tr>
<tr><td></td><td></td><td></td><td></td><td></td><td></td><td></td></tr>
</table>

<u>Additional File 1: Individual Survey</u>

SURVEY COMPONENT

<table>
<tr><td colspan="8">Demographics</td></tr>
<tr><td></td><td>Occupation</td><td>None
Student
Manual Work
Farmer/Fisherman
Civil Worker
Home Duties
Other: ___________________</td><td>1
2
3
4
5
6
8</td><td></td><td>Lived in this village for whole life?</td><td>No
Yes</td><td>0
1</td></tr>
<tr><td></td><td>Work Location</td><td>Indoor
Outdoor
Mixed</td><td>1
2
3</td><td></td><td colspan="2">If no, how many years?</td><td></td></tr>
</table>

<table>
<tr><td colspan="8">Infection/Personal risk factors</td></tr>
<tr><td></td><td>Slept under a bed net last night?</td><td>No
Yes</td><td>0
1</td><td></td><td>How often do you wear shoes outside?</td><td>Never
Rarely
Sometimes
Most of the day
Always</td><td>0
1
2
3
4</td></tr>
<tr><td></td><td>How many times do you bathe with soap per week?</td><td colspan="2"></td><td></td><td>Wearing Shoes now?</td><td>No
Yes</td><td>0
1</td></tr>
<tr><td colspan="8">Infection/symptoms and morbidity</td></tr>
<tr><td></td><td>Have you ever been told you have LF?</td><td>No
Yes</td><td>0
1</td><td></td><td>Have you ever had treatment for LF?</td><td>No (skip to #)
Yes</td><td>0
1</td></tr>
<tr><td></td><td>If Yes, at what age?</td><td colspan="2"></td><td></td><td>If yes, what kind of treatment</td><td>Tablets
Surgery
Other</td><td>1
2
8</td></tr>
<tr><td></td><td>Have you ever noticed swelling of the limbs, breast or scrotum?</td><td>No (skip to #)
Yes</td><td>0
1</td><td rowspan="2"></td><td rowspan="2">Have you had an episode of dermatolymphangioadenitis (Sudden onset local tenderness and redness of the skin, red streaks along the leg and enlarged inguinal lymph nodes) in the last 6 months?</td><td rowspan="2">No (skip to #)
Yes</td><td rowspan="2">0
1</td></tr>
<tr><td></td><td>If Yes, what symptoms?</td><td colspan="2"></td></tr>
<tr><td></td><td>If Yes, at what age?</td><td colspan="2"></td><td></td><td>If yes, how many times in last 6 months?</td><td colspan="2"></td></tr>
<tr><td colspan="8">MDA Participation</td></tr>
<tr><td></td><td>Have you ever taken the medication for LF in a mass drug administration programme?</td><td>No
Yes</td><td>0
1</td><td></td><td>Did you have any side effects?</td><td>No
Yes</td><td>0
1</td></tr>
<tr><td></td><td>If yes, did you take the medication last year?</td><td>No
Yes</td><td>0
1</td><td></td><td>If yes, what where they?</td><td colspan="2"></td></tr>
<tr><td rowspan="2"></td><td rowspan="2">If yes, how many times had you take medication total?</td><td colspan="2" rowspan="2"></td><td></td><td>Have you ever been not eligible for MDA</td><td>No
Yes</td><td>0
1</td></tr>
<tr><td></td><td>If Yes, why?</td><td colspan="2"></td></tr>
</table>

နေ့စွဲ		နာမည် အတို		မြို့နယ်	ကျေးရွာ		အိမ်ခြေ		လူဦးရေ	

MORBIDITY COMPONENT

1. Lymphadenitis & Lymphangitis

	Lymphadenitis Present?	No<br>Yes	0<br>1
	If Yes, details<br>*Side: (Right/Left), Area: (Axilla/Inguinal)*<br>*Symptoms: (Redness/Tenderness)*		
	Lymphangitis (red streaks) Present?	No<br>Yes	0<br>1
	If Yes, details<br>*Location*<br>*Symptoms: (Redness/Tenderness)*		

2. Lymphoedema & Limb Elephantiasis

	Clinical Signs		R Arm	L Arm	R Leg	L Leg
	Limb Circumference *(6cm from the wrist; 10cm proximal to the lateral malleolus)*					
	Lymphoedema Present?	No<br>Yes	0<br>1	0<br>1	0<br>1	0<br>1
	If yes, Oedema Type	Pitting<br>Non-Pitting	0<br>1	0<br>1	0<br>1	0<br>1
	Stemmer Sign Positive	No<br>Yes	0<br>1	0<br>1	0<br>1	0<br>1
	If Lymphoedema, what stage?	Lymphoedema (pitting) reversible overnight<br>Irreversible (non-pitting) lymphoedema + normal skin<br>Stage 2 + thick skin and shallow folds with visible base<br>Stage 2 + knobs (bumps and lumps)<br>Stage 2 + deep folds with base visible when separated by finger;<br>Irreversible lymphoedema with mossy foot<br>Stage 2 + disability to do routine activities adequately.	1<br>2<br>3<br>4<br>5<br>6<br>7	1<br>2<br>3<br>4<br>5<br>6<br>7	1<br>2<br>3<br>4<br>5<br>6<br>7	1<br>2<br>3<br>4<br>5<br>6<br>7
	Other Details					

3. Hydrocoele and Scrotal Elephantiasis

			Right Testicle	Left Testicle
	Hydrocoele	No<br>Yes	0<br>1	0<br>1
	If Yes, Stage	Normal<br>Scrotum size < tennis ball vertically<br>Scrotum size > tennis ball, lower pole doesn't reach mid-thigh<br>Lower pole reaches below mid-thigh but above superior patella<br>Lower pole between superior patella and tibial tuberosity<br>Lower pole between tibial tuberosity and mid-leg<br>Lower pole below the mid-leg	0<br>1<br>2<br>3<br>4<br>5<br>6	0<br>1<br>2<br>3<br>4<br>5<br>6
	If Yes, Penis Burial	Normal<br>Partial burial, >2cm visible<br>Partial burial, <2cm visible<br>Only prepuce/glans visible<br>Complete burial	0<br>1<br>2<br>3<br>4	0<br>1<br>2<br>3<br>4
	Diameter on USG			
	Other details			

နေ့စွဲ		နာမည် အတို		မြို့နယ်	ကျေးရွာ	အိမ်ခြေ	လူဦးရေ

<u>**တစ်ဦးချင်းစီမေးရန်**</u>

SURVEY COMPONENT

Demographics							
၅၀	အလုပ်အကိုင် (Occupation)	အလုပ်မရှိ<br>ကျောင်းသား<br>အလုပ်သမား<br>လယ်သမား၊ ရေလုပ်သား<br>ဝန်ထမ်း Civil Worker<br>အိမ်မှုကိစ္စ<br>အခြား:______________	၁<br>၂<br>၃<br>၄<br>၅<br>၆<br>၈	၅၂	ဒီရွာမှာပဲမွေးကတည်းကနေတာလား (Lived in this village for whole life?)	မရှိ<br>ရှိ	၀<br>၁
၅၁	**အလုပ်အနေအထား/နေရာ**	အိမ်တွင်း<br>အိမ်ပြင်<br>အိမ်တွင်း/အိမ်ပြင်	၁<br>၂<br>၃	၅၃	မဟုတ်ပါက ဒီမှာနေတာဘယ်လောက်ကြာပြီလဲ (If no, how many years?)		

Infection/Personal risk factors							
၅၄	လွန်ခဲ့သောညက ခြင်ထောင်ဖြင့် အိပ်ခဲ့ခြင်း ရှိမရှိ (Slept under a bed net last night?)	မရှိ<br>ရှိ	၀<br>၁	၅၆	**မကြာခဏအပြင်ထွက်တိုင်း ဖိနပ်စီးရဲ့လား၊** (How often do you wear shoes outside?)	မစီးပါ<br>တစ်ခါတစ်ရံ<br>နေ့တစ်ချို့<br>နေ့တော်တော်များများ<br>နေ့တိုင်း	၀<br>၁<br>၂<br>၃<br>၄
၅၅	တစ်ပတ်မှာ ဘယ်နှစ်ရက် ဆပ်ပြာနဲ့ရေချိုးသလဲ (Bathe Freq.?)			၅၇	အခုလောလောဆယ် ဖိနပ်စီးထားလား (Shoes?)	မရှိ<br>ရှိ	၀<br>၁
Infection/symptoms and morbidity **မေးခွန်း (၅၈)တွင် မရှိပါက မေးခွန်း (၆၇)သို့ သွားပါ။**							
၅၈	ဆင်ခြေထောက်ရောဂါ ရှိကြောင်း အသိပေးခံရဖူးပါသလား (Told you have LF?)	မရှိ<br>ရှိ	၀<br>၁	၆၃	ဆင်ခြေထောက်ရောဂါအတွက် ဆေးကုသမှု ခံယူဖူးပါသလား (Treatment for LF?)	မရှိ<br>ရှိ	၀<br>၁
၅၉	ရှိခဲ့လျှင် မည်သည့်အရွယ်က ဖြစ်ခဲ့သလဲ (At what age?)			၆၄	ရှိခဲ့လျှင်၊ မည့်သည့်ကုသမှုမျိုး ရရှိခဲ့ပါသလဲ (Type of Treatment)	ဆေးပြားသောက်<br>ခွဲစိတ်<br>အခြား	၀<br>၁<br>၈
၆၀	သင်၏ ခြေလက်၊ ရင်သား၊ ကပ်ပယ်အိတ်များတွင် ယောင်ယမ်းခြင်း ရှိခဲ့ခြင်းကို သတိပြုမိခဲ့ပါလား (LF Symptoms)	မရှိ<br>ရှိ	၀<br>၁	၆၅	လွန်ခဲ့သော ၆ လအတွင်း အရေပြားနာကျင်ခြင်း၊ နီရဲခြင်း၊ ခြေထောက်တွင် အနီစင်းများပေါ်ခြင်း၊ ပေါင်ခြံတွင် အကျိတ်များ ရုတ်တရက်ပေါ်ခြင်း ရှိခဲ့ဖူးပါသလား (Episodes of ADLA)	မရှိ<br>ရှိ	၀<br>၁
၆၁	**တကယ်လို့ ရောဂါရှိနေဖြစ်နေချိန်ရော**						
၆၂	ရှိခဲ့လျှင် မည်သည့်အရွယ်က ဖြစ်ခဲ့သလဲ			၆၆	ရှိခဲ့လျှင် ပြီးခဲ့သည့် ၆လအတွင်း အကြိမ်မည်မျှ ဖြစ်ခဲ့ပါသလဲ (Freq.)		
MDA Participation							
၆၇	ဆင်ခြေထောက်ရောဂါအတွက် လူအားလုံးဆေးတိုက်ကျွေးခြင်းတွင် ဆေးသောက်ဖူးပါသလား	မရှိ<br>ရှိ	၀<br>၁	၇၀	ဆေး၏ ဘေးထွက်ဆိုးကျိုးများ ခံစားဖူးပါသလား (side effects?)	မရှိ<br>ရှိ	၀<br>၁
၆၈	**ပြီးခဲ့တဲ့နှစ်က ဆင်ခြေထောက်ကာကွယ်ဆေးသောက်ခဲ့သလား**	မရှိ<br>ရှိ	၀<br>၁	၇၁	**ခံစားရတယ်ဆိုရင်ဆိုးကျိုးတွေက ဘာတွေလဲ ၊**		
				၇၂	**ဆေးသောက်ရန် မသင့်တော်ဘူးလို သတ်မှတ်ခံရဖူးပါသလား**	မရှိ<br>ရှိ	၀<br>၁
၆၉	**သောက်ခဲ့တယ်ဆိုရင် စုစုပေါင်း ဘယ်နှစ်ကြိမ် သောက်ခဲ့သလဲ၊**			၇၃	**ဘာအကြောင်းကြောင့် မသောက်ရတာလဲ ၊** (If not, why?)		

နေ့စွဲ		နာမည် အတို		မြို့နယ်	ကျေးရွာ		အိမ်ခြေ		လူဦးရေ	

MORBIDITY COMPONENT

ပြန်ရည်/သားငန်ရည်ကျိတ်၊ ပြန်ရည်/သားငန်ရည်ကြောရောင်ရမ်းမှု

၇၄	Lymphadenitis Present?		မရှိ<br>ရှိ	၀<br>၁
၇၅	If Yes, details<br>*Side: (Right/Left), Area: (Axilla/Inguinal)*<br>*Symptoms: (Redness/Tenderness)*	ယာဘက် ဝဲဘက်		
၇၆	Lymphangitis (red streaks) Present?		မရှိ<br>ရှိ	၀<br>၁
၇၇	If Yes, details<br>*Location, Symptoms(Redness/ Tenderness)*			

ဆင်ခြေထောက်နှင့် ခြေ/လက်ကြီးမှု

	Clinical Signs			ယာဘက်လက်	ဝဲဘက်လက်	ယာဘက်ခြေထောက်	ဝဲဘက်ခြေထောက်
၇၈	Limb လုံးပတ် *(6cm from wrist; 10cm from lateral malleolus)* (စင်တီမီတာ)						
၇၉	ခြေ/လက်ရောင်ရမ်းမှု ?<br>(Limb oedema present?)		မရှိ<br>ရှိ	၀<br>၁	၀<br>၁	၀<br>၁	၀<br>၁
၈၀	ရှိခဲ့လျှင် ရောင်ရမ်းမှု အဆင့်<br>(Oedema Type)		Pitting<br>Non-Pitting	၀<br>၁	၀<br>၁	၀<br>၁	၀<br>၁
၈၁	Stemmer Sign Positive		မရှိ<br>ရှိ	၀<br>၁	၀<br>၁	၀<br>၁	၀<br>၁
၈၂	လက်ရောင်ရမ်းမှု အဆင့်သတ်မှတ်မှု<br>(Stages)		Lymphoedema reversible overnight<br>Irreversible lymphoedema + normal skin<br>Stage 2 + thick skin and shallow folds with visible base<br>Stage 2 + knobs (bumps and lumps)<br>Stage 2 + deep folds with base seen separated by finger;<br>Irreversible lymphoedema with mossy foot<br>Stage 2 + disability to do routine activities adequately.	၁<br>၂<br>၃<br>၄<br>၅<br>၆<br>၇	၁<br>၂<br>၃<br>၄<br>၅<br>၆<br>၇	၁<br>၂<br>၃<br>၄<br>၅<br>၆<br>၇	၁<br>၂<br>၃<br>၄<br>၅<br>၆<br>၇
၈၃	အသေးစိတ်ဖေါ်ပြမှု (Details)						

ရေမှုတ္တနှင့် ကပ်ပယ်အိတ်ရောင်ရမ်းမှု

			ကပ်ပယ် အိတ် ယာဘက်	ကပ်ပယ် အိတ် ဝဲဘက်
၈၄	ရေမှုတ္တ<br>(Stage)	မရှိ<br>ရှိ	၀<br>၁	၀<br>၁
၈၅	If Yes, Stage	Normal<br>Scrotum size < tennis ball vertically<br>Scrotum size > tennis ball, lower pole doesn't reach mid-thigh<br>Lower pole reaches below mid-thigh but above superior patella<br>Lower pole between superior patella and tibial tuberosity<br>Lower pole between tibial tuberosity and mid-leg<br>Lower pole below the mid-leg	၀<br>၁<br>၂<br>၃<br>၄<br>၅<br>၆	၀<br>၁<br>၂<br>၃<br>၄<br>၅<br>၆
၈၆	If Yes, Penis Burial	Normal<br>Partial burial, >2cm visible<br>Partial burial, <2cm visible<br>Only prepuce/glans visible<br>Complete burial	၀<br>၁<br>၂<br>၃<br>၄	၀<br>၁<br>၂<br>၃<br>၄
၈၇	USG တွင်တွေ့ရသောအရွယ်အစား (စင်တီမီတာ) (USS Diameter)			
၈၈	အသေးစိတ်ဖေါ်ပြမှု (Details)			

နေ့စွဲ		နာမည်အတို		မြို့နယ်	ကျေးရွာ		အိမ်ခြေ		လူဦးရေ	
