# Supplementary material for: Risk factors for lymphatic filariasis and mass drug administration non-participation in Mandalay Region, Myanmar
Source: Parasit Vectors. 2021 Jan 22;14:72. doi: 10.1186/s13071-021-04583-y (PMC7821648; doi:10.1186/s13071-021-04583-y)
Supplement: Supplementary file 2 — Additional file 2. Household survey in English and Myanmar languages. [file 13071_2021_4583_MOESM2_ESM.pdf]

Additional File 2: Household Survey

A: HOUSEHOLD IDENTIFICATION

**Date (dd/mm/yyyy)**				
**Interviewer (initials)**				
	Township	*Village*	*Household Number*	*Household Head Name*
**Household Details**				
**Household Code**				

	Latitude (00.00000)	*Longitude (000.00000)*	*Elevation (0000m)*
**Household Location**			

	Date (dd/mm/yyyy)	*Result Code*
**Visit 1**		
**Visit 2**		

Result Codes: 1= Completed, 2= Not Home, 3= Vacated, 4= Postponed, 5= Refused, 6=Other

B: HOUSEHOLD ENROLLMENT

**Total Persons in Household**								
**Respondent (Tick)**		**Initials**	**Age (years)**	**1+**	**15+**	**Sex**	**Present (tick)**	**Slept Here Last Night (tick)**
	1							
	2							
	3							
	4							
	5							
	6							
	7							
	8							
	9							
	10							

နေ့စွဲ		မြို့နယ်	ကျေးရွာ		အိမ်ခြေ	

ကျေးဇူးပြုပြီး မေးခွန်းလေးတွေမသိဘူးဆိုရင် “ ၉ ” လို့ရေးပေးပါ။

C: HOUSEHOLD QUESTIONNAIRE

Demographics							
**1**	Ethnicity	Bamar<br>Other:__________	1<br>8	**4**	Respondent Schooling	None<br>Primary<br>Secondary<br>Tertiary	1<br>2<br>3<br>4
**2**	Religion	Buddhist<br>Other:__________	1<br>8	**5**	Highest Schooling in Household	None<br>Primary<br>Secondary<br>Tertiary	1<br>2<br>3<br>4
**3**	Literacy (show sentence)	None<br>Part of Sentence<br>Whole Sentence<br>Other Language	1<br>2<br>3<br>8	**6**	Monthly Income of household		

Physical Household Details							
7	Roof Material	Thatch<br>Metal<br>Tiles<br>Other: ______________	1<br>2<br>3<br>8	**11**	Screens/ Glass on most windows	No<br>Yes	0<br>1
**8**	Wall Material	Rattan / Natural<br>Wood<br>Bricks/Cement<br>Other ______________	1<br>2<br>3<br>8	12	Number of bed-nets in the house?		
**9**	Floor Material	Natural<br>Wood Planks<br>Tiles, Cement, Polished Wood<br>Other ______________	1<br>2<br>3<br>8	13	Toilet Type	Open-defecation<br>Unimproved<br>Shared<br>Improved	1<br>2<br>3<br>4
**10**	Number of rooms in the house?			**14**	Water Type	Surface<br>Unimproved<br>Other Improved<br>Piped Water	1<br>2<br>3<br>4

ကျောင်းနေအရွယ် ကလေးတိုင်း ကျောင်းသွားရမယ်။

နေ့စွဲ		မြို့နယ်	ကျေးရွာ		အိမ်ခြေ	

ကျေးဇူးပြုပြီး မေးခွန်းလေးတွေမသိဘူးဆိုရင် “ ၉ ” လို့ရေးပေးပါ။

Principal Components Analysis Asset register							
15	Electricity	No<br>Yes	0<br>1	26	Mobile	No<br>Yes	0<br>1
16	Piped water	No<br>Yes	0<br>1	27	Radio	No<br>Yes	0<br>1
17	Indoor Toilet	No<br>Yes	0<br>1	28	TV	No<br>Yes	0<br>1
18	Indoor Shower	No<br>Yes	0<br>1	29	Computer	No<br>Yes	0<br>1
19	Fan	No<br>Yes	0<br>1	30	Generator	No<br>Yes	0<br>1
20	Air-conditioning	No<br>Yes	0<br>1	31	Refrigerator	No<br>Yes	0<br>1
21	Screens	No<br>Yes	0<br>1	32	Bicycle	No<br>Yes	0<br>1
22	Chickens	No<br>Yes	0<br>1	33	Animal drawn Cart	No<br>Yes	0<br>1
23	Cattle/Ox	No<br>Yes	0<br>1	34	Motorcycle	No<br>Yes	0<br>1
24	Goats	No<br>Yes	0<br>1	35	Car	No<br>Yes	0<br>1
25	Other animals:______________________			36	Boat	No<br>Yes	0<br>1

LF Knowledge Questions			
37	Have you heard about LF before the study?	No<br>Yes	0<br>1
38	Have you ever seen LF in your village? (show picture)	No<br>Yes	0<br>1
39	How is LF Transmitted? (show picture)	Walking barefoot<br>Water/Food<br>Injury<br>Sex<br>Air-borne<br>Mosquitos<br>Skin-Contact<br>Unclean Environment<br>Poor Hygiene<br>Witchcraft<br>Other<br>Don’t know	01<br>02<br>03<br>04<br>05<br>06<br>07<br>08<br>09<br>10<br>88<br>99

နေ့စွဲ		မြို့နယ်	ကျေးရွာ		အိမ်ခြေ	

ကျေးဇူးပြုပြီး မေးခွန်းလေးတွေမသိဘူးဆိုရင် “ ၉ ” လို့ရေးပေးပါ။

40	What are the symptoms of LF?		
41	How is LF Treated?	Tablets<br>Surgery<br>Physiotherapy<br>Hygiene Practices<br>Other<br>Don’t Know	1<br>2<br>3<br>4<br>8<br>9

Treatment and Mass Drug Administration Questions			
42	Had you heard about the National Programme to Eliminate LF before this study?	No<br>Yes	0<br>1
43	Has your household or village been visited by the LF mass drug administration programme and offered medication?	No<br>Yes	0<br>1
44	If yes, how many times?		
45	How many individuals were living in your household during the time of the last MDA? *(Including those not eligible, those who do not currently live in the household and those not present)*		
46	Have you or your family ever declined to take part in an LF mass drug administration?	No<br>Yes	0<br>1
47	If yes, why?		

အိမ်ခြေ စစ်တမ်းကောက်ယူခြင်း (အိမ်ထောင်ဦးစီးအားမေးရန်) (HH Representative)

A: အိမ်ခြေအမှတ်အသား

နေ့စွဲ **(dd/mm/yyyy)**				
မေးမြန်းသူ **(လက်မှတ်)**				
	မြို့နယ်	ကျေးရွာ	အိမ်ခြေ	အိမ်ထောင် ဦးစီးအမည်
အိမ်ခြေ **Details**				
အိမ်ခြေ**CODE**				
	Latitude (00.00000)	*Longitude (000.00000)*	*Elevation (0000m)*	
**အိမ်နေရာအနေအထား**				
	နေ့စွဲ *(dd/mm/yyyy)*	အခြေအနေ		
**အလည်အပတ်**–(၁)		၁ ၂ ၃ ၄ ၅ ၆		
**အလည်အပတ်**–(၂)		၁ ၂ ၃ ၄ ၅ ၆		

အခြေအနေ**CODE:** ၁။ ပြီးစီး ၂။ အိမ်မှာမရှိ ၃။ ယာယီရွှေ့ပြောင်း ၄။ နောက်တခေါက် ထပ်လာရန် ၅။ ငြင်းဆို ၆။ အခြား

B:မိသားစုစာရင်း

လူဦးရေ စုစုပေါင်း									
ဖြေဆိုသူကို အမှတ်အသားလုပ်ရန် (√)		နာမည် အတို	အသက်	၁**+**	၁၅ **+**	လိင်		အိမ်မှာရှိသူကို အမှတ်အသားလုပ်ရန် (√)	မနေ့ညက အိမ်မှာ အိပ်သလား (√)
						ကျား **M**	မ **F**		
☐	၁			☐	☐			☐	☐
☐	၂			☐	☐			☐	☐
☐	၃			☐	☐			☐	☐
☐	၄			☐	☐			☐	☐
☐	၅			☐	☐			☐	☐
☐	၆			☐	☐			☐	☐
☐	၇			☐	☐			☐	☐
☐	၈			☐	☐			☐	☐
☐	၉			☐	☐			☐	☐
☐	၁၀			☐	☐			☐	☐

နေ့စွဲ		မြို့နယ်	ကျေးရွာ		အိမ်ခြေ	

ကျေးဇူးပြုပြီး မေးခွန်းလေးတွေမသိဘူးဆိုရင် “ ၉ ” လို့ရေးပေးပါ။

C: HOUSEHOLD QUESTIONNAIRE

Demographics							
၁	လူမျိုး<br>(Ethnicity)	ဗမာ<br>အခြား:__________	၁<br>၈	၄	ဖြေဆိုသူအတန်း<br>(Respondent Schooling)	ကျောင်းမနေဖူးပါ<br>မူလတန်း<br>အလယ်တန်း၊အထက်တန်း<br>တက္ကသိုလ်	၁<br>၂<br>၃<br>၄
၂	ကိုးကွယ်သည့်ဘာသာ<br>(Religion)	Buddhist<br>အခြား:__________	၁<br>၈	၅	အိမ်သားများ၏အမြင့်ဆုံးအတန်း<br>(Highest Schooling in Household)	ကျောင်းမနေဖူးပါ<br>မူလတန်း<br>အလယ်တန်း၊အထက်တန်း<br>တက္ကသိုလ်	၁<br>၂<br>၃<br>၄
၃	စာဖတ်တတ်မတတ်<br>(အောက်မှစာကို ဖတ် ခိုင်းရန်)<br>Literacy	စာမတတ်<br>တလုံးနှစ်လုံးဖတ်တတ်၊<br>ဖတ်တတ်စာတတ်<br>တခြားဘာသာစကားတတ်လား	၁<br>၂<br>၃<br>၈	၆	မိသားစုအားလုံးပေါင်း၏တစ်လပျမ်းမျှဝင်ငွေ<br>(Monthly Income of household)		

Physical Household Details							
၇	အမိုး<br>(Roof Material)	သက်ကယ်<br>သွပ်<br>အုတ်ကြွပ်<br>အခြား:__________	၁<br>၂<br>၃<br>၈	၁၁	ခြင်မဝင်အောင်ပြတင်းပေါက်တွေကို တစ်ခုခု နဲ့ကာထားသလား (ခြင်ကာ၊ မှန်၊ သစ်သားပြတင်းကာ)<br>(Screens/ Glass on most windows)	မရှိ<br>ရှိ	၀<br>၁
၈	နံရံ<br>(Wall Material)	ဝါးထရံ<br>သစ်သား<br>အုတ်အင်္ဂတေ / ဘိလပ်မြေ/ အင်္ဂတေ<br>အခြား: __________	၁<br>၂<br>၃<br>၈	၁၂	ခြင်ထောင် ဘယ်နှစ်လုံးရှိသလဲ<br>(Number of bed-nets in the house?)		
၉	ကြမ်းခင်း<br>(Floor Material)	None<br>သစ်သား<br>ကြွေပြား /ဘိလပ်မြေ/ အင်္ဂတေ / အရောင်တင်သစ်သား/ သပ်သပ်ရပ်ရပ် }<br>အခြား:__________	၁<br>၂<br>၃<br>၈	၁၃	Toilet Type	Open-defecation<br>Unimproved<br>Shared<br>Improved	၁<br>၂<br>၃<br>၄
၁၀	အခန်းဘယ်နှစ်ခန်း ရှိသလဲ<br>(Number of rooms in the house?)			၁၄	Water Type	Surface<br>Unimproved<br>Other Improved<br>Piped Water	၁<br>၂<br>၃<br>၄

ကျောင်းနေအရွယ် ကလေးတိုင်း ကျောင်းသွားရမယ်။

နေ့စွဲ		မြို့နယ်	ကျေးရွာ		အိမ်ခြေ	

ကျေးဇူးပြုပြီး မေးခွန်းလေးတွေမသိဘူးဆိုရင် “ ၉ ” လို့ရေးပေးပါ။

Principal Components Analysis Asset register							
၁၅	လျှပ်စစ်မီး<br>(Electricity)	မရှိ<br>ရ	၀<br>၁	၂၆	မိုဘိုင်းဖုန်း<br>(Mobile)	မရှိ<br>ရှိ	၀<br>၁
၁၆	အိမ်နှင့်တွဲလျက်အိမ်သာ<br>(Indoor Toilet)	မရှိ<br>ရှိ	၀<br>၁	၂၇	ရေဒီယို<br>(Radio)	မရှိ<br>ရှိ	၀<br>၁
၁၇	ရုပ်မြင်သံကြားစက်<br>(TV)	မရှိ<br>ရှိ	၀<br>၁	၂၈	လေအေးပေးစက်<br>(Air conditioning)	မရှိ<br>ရှိ	၀<br>၁
၁၈	အိမ်တွင်းရေချိုးခန်း<br>(Indoor Shower)	မရှိ<br>ရှိ	၀<br>၁	၂၉	Computer	မရှိ<br>ရှိ	၀<br>၁
၁၉	မျက်နှာကျက်ပန်ကာ<br>(Fan)	မရှိ<br>ရှိ	၀<br>၁	၃၀	Generator	မရှိ<br>ရှိ	၀<br>၁
၂၀	ခြင်စကာပြတင်းပေါက်အားလုံး<br>(Screens on windows)	မရှိ<br>ရှိ	၀<br>၁	၃၁	ရေခဲသေတာ<br>(Refrigerator)	မရှိ<br>ရှိ	၀<br>၁
၂၁	စက်ဘီး<br>(Bicycle)	မရှိ<br>ရှိ	၀<br>၁	၃၂	ပိုက်လိုင်းရေပေးဝေမှု<br>(Piped water)	မရှိ<br>ရှိ	၀<br>၁
၂၂	ကြက်<br>(Chickens)	မရှိ<br>ရှိ	၀<br>၁	၃၃	နွားလှည်း<br>(Animal drawn Cart)	မရှိ<br>ရှိ	၀<br>၁
၂၃	နွား ကျွဲ<br>(Cattle/Ox)	မရှိ<br>ရှိ	၀<br>၁	၃၄	မော်တော်ဆိုင်ကယ်<br>(Motorcycle)	မရှိ<br>ရှိ	၀<br>၁
၂၄	ဆိတ်<br>(Goats)	မရှိ<br>ရှိ	၀<br>၁	၃၅	ကား<br>(Car)	မရှိ<br>ရှိ	၀<br>၁
၂၅	အခြားတိရစ္ဆာန်			၃၆	လှေ<br>(Boat)	မရှိ<br>ရှိ	၀<br>၁

LF Knowledge Questions			
၃၇	အရင်ကဆင်ခြေထောက်ရောဂါအကြောင်းကြားဖူးပါသလား<br>(Have you heard about LF before the study?)	မရှိ<br>ရှိ	၀<br>၁
၃၈	သင်နေထိုင်ရာကျေးရွာတွင် ဆင်ခြေထောက်ရောဂါဖြစ်နေသူတွေ့ဖူးပါသလား<br>(Have you ever seen LF in your village? (show picture))	မရှိ<br>ရှိ	၀<br>၁

၃၉	ဆင်ခြေထောက်ရောဂါကဘယ်လိုကူးစက်ပါသလဲ<br>(How is LF Transmitted?)	ဖိနပ်မပါဘဲလမ်းလျှောက်လို့<br>အစားမှားလို့ကူးတာ/ရေကကူးတာ<br>ထိခိုက်မိလို့<br>လိင်မှုတဆင့်ကူးဆက်<br>လေထဲကကူးတာ<br>ခြင်ကိုက်လို့<br>အရေပြားထိတွေ့မှုမှတဆင့် ကူးစက်<br>ပတ်ဝန်းကျင်မသန့်လို့<br>တကိုယ်ရေသန့်ရှင်းမှု မရှိ<br>စုန်းပြုစားလို့<br>အခြား________________<br>မသိပါ	၀၁<br>၀၂<br>၀၃<br>၀၄<br>၀၅<br>၀၆<br>၀၇<br>၀၈<br>၀၉<br>၁၀<br>၈၈<br>၉၉

နေ့စွဲ		မြို့နယ်	ကျေးရွာ		အိမ်ခြေ	

ကျေးဇူးပြုပြီး မေးခွန်းလေးတွေမသိဘူးဆိုရင် “ ၉ ” လို့ရေးပေးပါ။

၄၀	ဆင်ခြေထောက်ရောဂါလက္ခဏာများ<br>(Symptoms of LF?)	

၄၁	ဆင်ခြေထောက်ရောဂါကိုဘယ်လိုကုရသလဲ။<br>(How is LF Treated?)	ဆေးပြားသောက်<br>ခွဲစိတ်<br>လေ့ကျင့်ခန်းလုပ်<br>တစ်ကိုယ်ရည်သန့်ရှင်းရေး<br>အခြား<br>မသိပါ	၁<br>၂<br>၃<br>၄<br>၈<br>၉

လူအားလုံးဆေးကျွေးခြင်းနှင့်ဆေးကုသမှု			
၄၂	ဆင်ခြေထောက်ရောဂါကင်းဝေးရေး စီမံချက်ကို အရင်ကကြားဖူးသလား<br>(Had you heard about the National Programme to Eliminate LF before this study?)	မရှိ<br>ရှိ	၀<br>၁
၄၃	သင်တို့နေထိုင်ရာရပ်ရွာကို ဆင်ခြေထောက်ရောဂါကာကွယ်ဆေး ကွင်းဆင်းတိုက်ကျွေးပေးသောအဖွဲ့ ရှိလား၊<br>(Household/Village visited by the LF MDA and offered medication?)	မရှိ<br>ရှိ	၀<br>၁
၄၄	ရှိရင်ဘယ်နှစ်ကြိမ်လာသလဲ၊		
၄၅	ဆေးလာတိုက်တဲ့အချိန် အိမ်မှာလူဘယ်နှစ်ယောက်ရှိလဲ<br>(How many individuals living in household during last MDA?)		
၄၆	လူအားလုံးဆေးကျွေးစဉ်က သင့်နှင့် သင့်မိသားစု ဆေးသောက်ရန်ငြင်းဆိုဖူးသလား။<br>(Have you or your family ever declined to take part in an LF mass drug administration?)	မရှိ<br>ရှိ	၀<br>၁
၄၇	ငြင်းဖူးတယ်ဆိုရင် ဘာကြောင့်လဲ၊<br>(If yes, Why?)		
