# Supplementary figures and images for: Risk factors for lymphatic filariasis and mass drug administration non-participation in Mandalay Region, Myanmar
Source: Parasit Vectors. 2021 Jan 22;14:72. doi: 10.1186/s13071-021-04583-y (PMC7821648; doi:10.1186/s13071-021-04583-y)

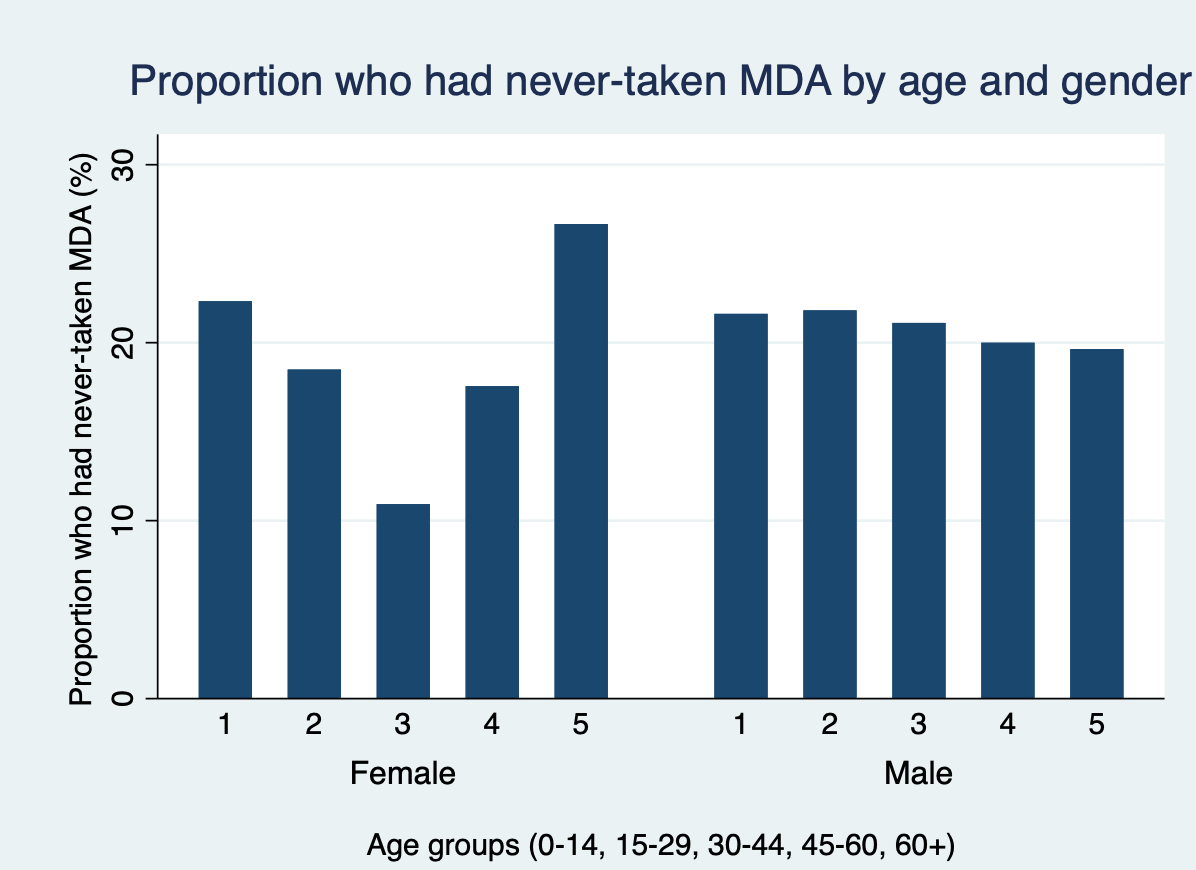

Supplement: Supplementary file 3 — Additional file 3: Figure S1. Unadjusted proportion who never took MDA by age and gender. [file 13071_2021_4583_MOESM3_ESM.png]

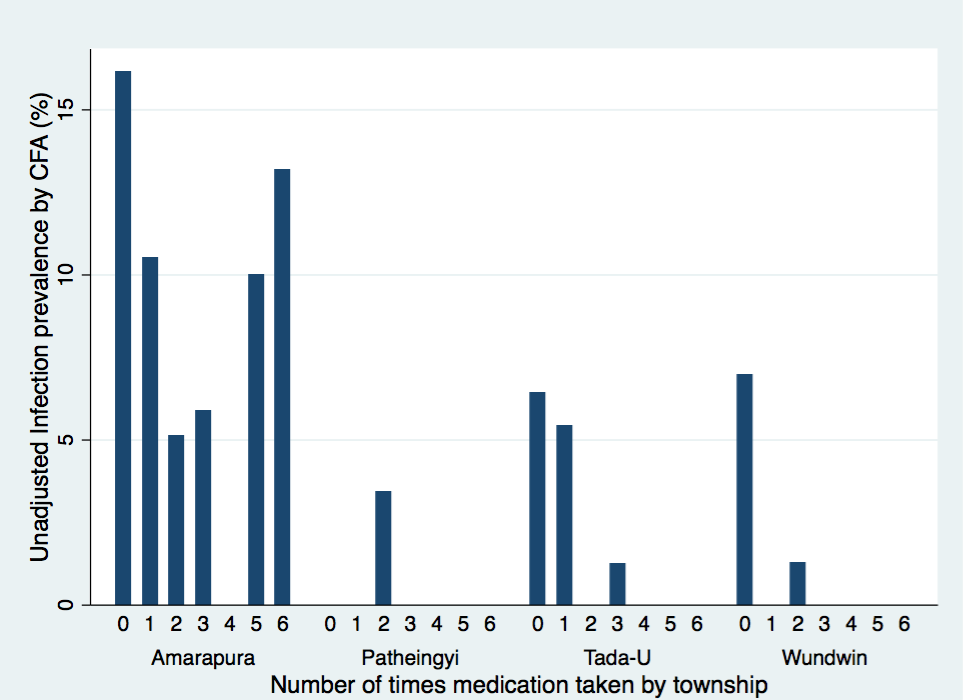

Supplement: Supplementary file 4 — Additional file 4: Figure S2. Unadjusted infection prevalence by number of times MDA medication taken and township. [file 13071_2021_4583_MOESM4_ESM.png]
